# Identification of the Olfactory Profile of Rapeseed Oil as a Function of Heating Time and Ratio of Volume and Surface Area of Contact with Oxygen Using an Electronic Nose

**DOI:** 10.3390/s21010303

**Published:** 2021-01-05

**Authors:** Robert Rusinek, Dominik Kmiecik, Marzena Gawrysiak-Witulska, Urszula Malaga-Toboła, Sylwester Tabor, Pavol Findura, Aleksander Siger, Marek Gancarz

**Affiliations:** 1 Institute of Agrophysics Polish Academy of Sciences, Doświadczalna 4, 20-290 Lublin, Poland; 2 Department of Gastronomy Science and Functional Food, Faculty of Food Science and Nutrition, Poznan, University of Life Sciences, Wojska Polskiego 31, 60-634 Poznan, Poland; dominik.kmiecik@up.poznan.pl; 3 Institute of Food Technology of Plant Origin, Faculty of Food Science and Nutrition, Poznan University of Life Science, Wojska Polskiego 28, 60-637 Poznan, Poland; marzena.gawrysiak-witulska@up.poznan.pl; 4 Faculty of Production and Power Engineering, University of Agriculture in Krakow, Balicka 116B, 30-149 Krakow, Poland; umalagatobola@gmail.com (U.M.-T.); Sylwester.Tabor@urk.edu.pl (S.T.); marekgancarz74@gmail.com (M.G.); 5 Department of Machines and Production Biosystems, Faculty of Engineering, Slovak University of Agriculture in Nitra, Hlinku 2, 949 76 Nitra, Slovakia; pavol.findura@uniag.sk; 6 Department of Food Biochemistry and Analysis, Faculty of Food Science and Nutrition, Poznań University of Life Sciences, Wojska Polskiego 31, 60-634 Poznan, Poland; asiger@up.poznan.pl

**Keywords:** rapeseed oil, electronic nose, gas chromatography, polar compounds, frying

## Abstract

The process of deep fat frying is the most common technological procedure applied to rapeseed oil. During heat treatment, oil loses its nutritional properties and its original consumer quality is lowered, which is often impossible to determine by organoleptic assessment. Therefore, the aim of the study was to correlate markers of the loss of the nutritional properties by rapeseed oil related to the frying time and the surface area of contact with oxygen with changes in the profile of volatile compounds. The investigations involved the process of 6-, 12-, and 18-h heating of oil with a surface-to-volume ratio (s/v ratio) of 0.378 cm^−1^, 0.189 cm^−1^, and 0.126 cm^−1^. Samples were analysed to determine changes in the content of polar compounds, colour, fatty acid composition, iodine value, and total chromanol content. The results were correlated with the emission of volatile compounds determined using gas chromatography and an electronic nose. The results clearly show a positive correlation between the qualitative degradation of the oil induced by prolonged heating and the response of the electronic nose to these changes. The three volumes, the maximum reaction of the metal oxide semiconductor chemoresistors, and the content of polar compounds increased along the extended frying time.

## 1. Introduction

Plant oils are the main source of unsaturated fatty acid and other substances, e.g., tocopherols and phytosterols, in the human diet. Unsaturated fatty acids are very important for human health. They play a key role in the structure of membranes, the synthesis of eicosanoids, and the transport and metabolism of cholesterol, reducing its concentration in the blood plasma. They are essential for prevention of the progression of cardiovascular disease (CVD) and for development of the brain, especially during pregnancy and early life. Unsaturated fatty acids may increase tissue sensitivity to insulin, which is important for patients with carbohydrate metabolism disorders, e.g., diabetes. Additionally, they exert anti-cancer, anti-inflammatory, and immunomodulatory effects [1,2,3]. Tocopherols contained in oil play an important role as vitamins and antioxidants in the human body [4]. Phytosterols can significantly contribute to reduction of the level of low-density lipoprotein (LDL) cholesterol in human blood [5].

Oxidation is one of the most important reactions that can affect the sensory quality and nutritional value of plant oils. The main oil compounds that undergo oxidation are fatty acids, which contain unsaturated bonds in their structure. Other unsaturated compounds present in vegetable oils, such as tocopherols and phytosterols, are susceptible to oxidation as well [6,7].

Oxidation is the main chemical reaction observed during oil storage and in the oil heating/frying process. It occurs in oils under the influence of oxygen from the air. However, the process of hydrolysis and thermal changes are also observed in oil exposed to high temperature and moisture (from the fried product) [8]. These three reactions taking place in heated oil lead to emergence of two types of new components (low and high molecular weight). The first group of compounds with lower molecular weight, compared to parent triacylglycerols, comprises oxidised fatty acids, aldehydes, ketones, hydrocarbons, alcohols, or epoxides. The other group consists of compounds with higher molecular weight than that of parent triacylglycerols, i.e., dimmers, trimers, and oligomers of triacylglycerols [9]. The latter group of compounds, i.e., volatile substances, has a considerable impact on the quality and sensory characteristics of heated oil and fried food [10]. High content of polymers reduces the heat transfer process and increases the viscosity of oil and fat uptake in food. These compounds can also affect human health by increasing oxidative stress in the intestines, affect the blood lipid profile, or induce peroxidation of lipids in the human body [11,12]. The rate of quality changes observed in oil exposed to high temperature depends on many factors. They are related to the conditions of the frying process, the type of the oil used, and the type of the fried product [13,14].

The frying process leads to generation of a large group of volatile compounds through a number of different chemical reactions. They derive from two main sources. Low molecular weight compounds can be generated via oxidation of fatty acids and the Maillard reaction [15]. On the one hand, they positively contribute to the unique sensory characteristics of fried food; on the other hand, however, they are responsible for undesirable sensory traits. Additionally, the oxidation process and the Maillard reaction lead to formation of health-hazardous compounds. The nature of these compounds depends on several factors, mainly the fatty acid profile and susceptibility of oil to oxidation processes, the food components (content of protein and carbohydrates), the conditions and duration of the process (in particular oxygen access and time), and the ratio of the surface area of oil in contact with oxygen to its volume. Hence, a question arises whether volatile compounds can be a marker of the physicochemical state of oils.

There are many methodologies for assessment of the quality of food materials, also such liquid materials as rapeseed oil, e.g., organoleptic, instrumental, and chemical methods [16,17]. One of the recent widely used instrumental approaches is the assessment of quality based on the analysis of volatile substances [18]. In this field, gas chromatography is one of the most precise techniques for quality evaluation [19]. However, the dynamic development of the technology of production of electrochemical transducers and techniques for analysis of large data sets noted for several decades has increased the frequency of application of electronic noses to analyse volatile substances in many areas of life, including assessment of food quality [20]. The term “electronic nose” defines an instrument consisting of a set of chemically sensitive sensors that are able to recognise simple and complex odours and save them digitally in the virtual memory of the device [21]. The aim of the present study was to use an electronic nose for detection of volatile substances emitted from oils during long-term heat treatment of samples with different volumes and a constant surface area of contact with oxygen. Another objective was to correlate these sensor responses with the presence of characteristic volatile compounds. The analyses were carried out in a 6-, 12-, and 18-h oil heating process at a surface/volume (s/v) ratio of 0.378 cm^−1^, 0.189 cm^−1^, and 0.126 cm^−1^, which reflects the real process of frying food products. The investigations were conducted with the use of the Agrinose, i.e., a device with a measuring system based on a matrix of eight metal oxide semiconductor (MOS) sensors. This non-invasive and inexpensive device is used for rapid identification of the volatile compound profile [22]. The results of the measurements carried out with the electronic nose were verified and correlated with the more accurate but more labour-intensive and costly GC-MS analysis of volatile substances and chemical methods for determination of quality [23]. This was aimed at answering the question whether the history of heat treatment of oil can be revealed by the analysis of its volatile substances.

## 2. Materials and Methods

### 2.1. Materials

The raw material used in the study was the regular refined rapeseed oil (RRO) purchased shortly before the start of the experiments. Before the experiment, the oil was stored at 5 °C and protected from light. Unheated rapeseed oil was characterized using gas (GC-FID) and liquid (HPLC-FLD) chromatography techniques. The fatty acid profile and the total chromanol content were determined.

### 2.2. Heating Process

Before the heating process, the oil was allowed to stand at room temperature until a constant temperature was achieved by all samples. All oil samples were heated at 170 °C ± 5 °C in glass beakers with the same diameter and protected from light for 6, 12, and 18 h. The oil was heated in three volumes: 150 mL, 300 mL, and 450 mL yielding three different surface-to-volume ratios: 0.378 cm^−1^, 0.189 cm^−1^, and 0.126 cm^−1^, respectively. Magnetic stirrers (IKA RET basic, MS-H-Pro, IKA Works, Inc. Wilmington, NC, USA) and an electronic thermometer were used to heat the oil samples. The heated samples were stored under nitrogen atmosphere at −24 °C. All heating processes were carried out in two parallel replications.

### 2.3. Total Polar Compound (TPC) Analysis

The content of total polar compounds of oil was determined according to the American Oil Chemists’ Society-AOCS Official Method 982.27 [24]. Briefly, an oil sample was mixed with toluene and placed on a chromatography column packed with silica gel containing 5% of distilled water (silica gel 60, 63–200 µm, Sigma-Aldrich, Poznan, Poland). A mixture of hexane and diisopropyl ether (82:18, *v*:*v*) was used for elution of the non-polar fraction of oil. After the evaporation of the solvent, the non-polar fraction was weighed and the mass was used for calculation of the polar fraction. The polar fraction was calculated as the difference between the mass of the analysed sample and the mass of the non-polar fraction and expressed in %.

### 2.4. Iodine Value Calculation (CIV)

The degree of unsaturation (iodine value) of fresh oils and its changes during the heating process was calculated according to the AOCS Official Method Cd 1c-85 [25]. The calculation was done directly from fatty acid compositions according to the potential number of iodine atoms added to each triglyceride.

### 2.5. Fatty Acid Composition Analysis

Fatty acid composition analysis of fresh and heated samples was done according to the AOCS Official Method Ce 1h-05 [26,27]. Briefly, rapeseed oil was dissolved in *n*-hexane and transesterification with sodium methoxide was carried out. After transesterification, fatty acid methyl esters (FAME) were transferred to glass vials and analysed using gas chromatographic techniques. An Agilent 7820A GC gas chromatography system (Agilent Technologies, Santa Clara, CA, USA) equipped with a flame ionisation detector (FID) and a SLB-IL111 capillary column (100 m, 0.25 mm, 0.20 μm, Supelco, Bellefonte, PA, USA) were used for identification of individual fatty acids.

### 2.6. Tocopherol and Plastochromanol-8 Analysis

The contents of tocopherols and plastochromanol-8 (PC-8) were determined according to Siger et al. [25]. Rapeseed oil (0.2 g) was dissolved in *n*-hexane, made up to 10 mL, and transferred to vials for analyses. The identification and quantification of tocopherols and PC-8 were conducted on a Waters 600 Asc HPLC system (Waters, Milford, MA, USA) in normal phase (NP) mode using a LiChrosorb Si 60 column (250 × 4.6 mm, 5 μm, Merck KGaA, Darmstadt, Germany) and a fluorimetric detector. The mobile phase comprised a mixture of *n*-hexane with 1,4-dioxane (96:4 *v*/*v*). The flow rate was 1.0 mL/min. The fluorimetric detector (Waters 474 Asc.) was run at excitation λ = 290 nm and emission λ = 330 nm for tocopherols and PC-8.

### 2.7. Imaging Colorimeter

Changes in the oil colour were assessed according to the procedure described in Rusinek et al., [17]. Colour change tests were performed using a Lovibond CAM-System 500 colorimeter (city, UK). The calculation procedure used is described in the Supplementary Materials A (Section 2. Imaging colorimeter).

### 2.8. Electronic Nose

The Agrinose electronic nose designed and manufactured at the Institute of Agrophysics PAS (Lublin, Poland) was used for the research [17,28]. The technical data of the e-nose and the measurement procedure are described in the Supplementary Materials A (Section 1. Electronic nose procedure).

### 2.9. Gas Chromatography-Mass Spectrometry (GC-MS) Analysis

The basic steps of the experiment were as follows. The GC-MS analyses were carried out following the procedure described by Marek et al. [29]. A Trace GC Ultra gas spectrometer and an ITQ 1100 mass spectrometer were used as tools. Both devices were purchased from ThermoFisher Scientific (Waltham, MA, USA).

Before starting the tests, the column was calibrated with (C5-C24) (Supelco, Merck KGaA). The mixture of *n*-alkanes was analyzed for 1 μL of *n*-alkanes solution and analyzed. Alkane retention times obtained during the analysis were used to identify 138 tested standards in the Wiley library for the ZB-5MSplus Capillary GC 30 m × 0.25 mm × 0.25 μm column used. Such tests were performed because times could have varied due to, for example, poor column quality or the use of different gases as carriers therein. The obtained pattern retention times allowed for over 90% identification of the tested patterns in the Wiley 138 library, which was also used to identify the smells of the tested oil samples. The analyses were performed with programmed temperature: initial temperature 60 °C maintained for 5 min, from 60 to 250 °C at 5 °C/min, 250 to 270 °C at 10 °C/min, the final temperature being maintained for 5 min. The helium flow rate was held constant at 2.2 mL/min. The transfer line, ion source and quadrupole temperatures were 280 °C. Electron Ionization (EI_+_) mode with an electron energy of 70 eV was used. The mass spectrometer acquired data in full scan mode (scan ranges: 35–390). The compounds emitted from the surface of the tested samples adsorbed on the fibers were tested under the same conditions as for the standards. The test sample components were then identified using the obtained retention times and use of the Wiley 138 library.

The tested chemical compounds in the volatile phase were collected by divinylbenzene/carboxen/polydimethylsiloxane SPME fibres purchased from Sigma Aldrich. The fibre was placed for 30 min over the sample in the measurement chamber. Then, it was transferred into the GC-MS device. More technical information and procedure steps were described in another publication by the authors [26] and in the Supplementary Materials A (Section 3. Gas chromatography-mass spectrometry (GC-MS) analysis).

### 2.10. Principal Component Analysis (PCA)

The principal component analysis was performed using the Statistica software (version 12.0, StatSoft Inc., Tulsa, OK, USA) at the significance level α = 0.05. The Cattel criterion was used to obtain the optimal number of principal components. A data matrix with 29 columns and 10 rows was designed to assess the ability of the electronic nose to describe the odour profiles of the oil. The data was scaled automatically.

## 3. Results and Discussion

### 3.1. Total Polar Compound Content (TPC) and Fatty Acid Composition

**Figure 1:**
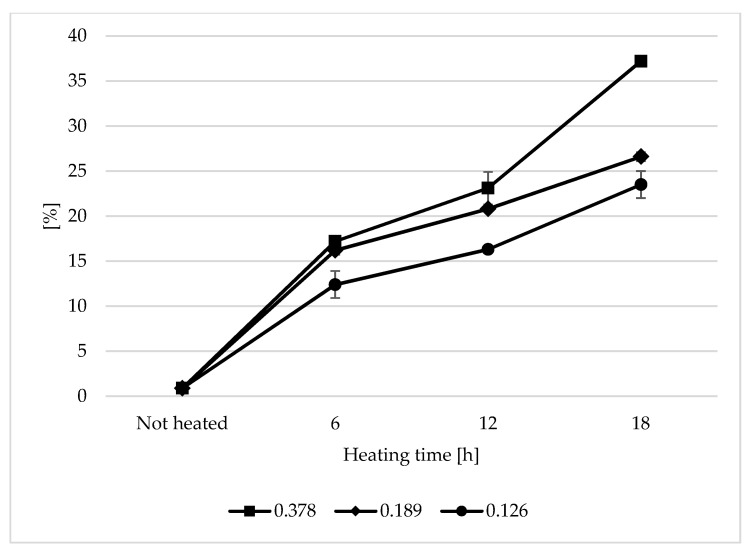
Changes in total polar compounds (TPC) during thermal treatment of refined (RRO) rapeseed oil with different surface-to-volume of oil ratios [%].

The content of total polar compounds is currently one of the best indicators showing the total changes occurring in oil used for frying. During the heating of the analysed oils, a steady increase in TPC was observed (Figure 1). The highest increase in TPC in the analysed samples was observed during the first 6 h of heating. The TPC content ranged from 46.24% to 60.9% of the final level of polar compounds. The largest increase in the TPC content was observed in the sample with the 0.378 s/v ratio, whereas the smallest change was noted at the s/v ratio of 0.126. After 18 h of heating, the polar content was 23.5%, 26.6%, and 37.2% for the heated oil samples with the s/v ratio of 0.126, 0.189, and 0.378, respectively. Assuming a level of 25% as the limit content of the polar fraction in frying oils [27], the only oil that did not exceed the assumed value was the sample with the 0.126 s/v ratio. The high content of the polar fraction was typical when the s/v ratio was 0.378. The value was by 49% higher than the limit. The increase in the polar fraction content is a typical phenomenon observed during the heating of vegetable oils; it occurs particularly quickly at high temperatures [28,29]. Alzaa et al. obtained similar results as those for samples heated with a ratio of 0.378 and 0.189 after 6 h of the thermal treatment. The content of the polar fraction for the ratio of 0.126 was lower than in the cited work. This proves the influence of the size of the oil contact surface with air on the oil degradation rate. Alzaa et al. also considered the composition of the tested oils and temperature of the heating process. The high content of unsaturated fatty acids and antioxidants plays an important role in the speed of oil degradation. By increasing the frying temperature, the authors observed a sharp increase in the polar fraction content. This was explained by an increase in thermal oxidation and polymerization of not only fatty acids but also other substances of the unsaponifiable fraction of oils, such as tocopherols. As a result of fatty acid degradation, many low and high molecular weight compounds are formed, which are most often polar compounds [30,31]. The rate of the degradation process is mainly dependent on the access of oxygen, the thickness of the oil layer, and the oil volume. The content of natural antioxidants—tocopherols—also played an important role. As the oil surface area was constant in the research and its mass (layer thickness) was variable, the initial tocopherol content in the sample changed. The content of tocopherols in the unheated oil was 82.14 mg/100 g (Table 1). In the heated samples with the s/v ratio of 0.378, a very sharp decrease in the tocopherol content was observed during the first 6 h of heating (95%), which indicates rapid oxidative processes occurring during the thermal treatment. In the other samples, the decrease was 62.7% (0.189 s/v ratio) and 39.9% (0.126 s/v ratio). The final tocopherol content was 0.33 mg/100g, 1.42 mg/100g, and 2.8 mg/100g for the heated oil with the 0.378, 0.189 and 0.126 s/v ratios, respectively.

The change in the surface-to-volume ratio of the heated oil also led to changes in the composition of fatty acids in the samples (Table 1). Saturated and monounsaturated fatty acids (MUFA) were observed to increase their contribution to all fatty acids. There was a decrease in the level of polyunsaturated fatty acids (PUFAs) in the pool of fatty acids and in the iodine value expressing the oil unsaturation degree. As before, PUFAs were degraded at highest rate when thin layers of oils were heated (0.378 s/v ratio). The increase in the volume of the heated oil (decrease in the surface-to-volume ratio) led to slower degradation of fatty acids. The decrease in the PUFA content was 7.59, 4.66, and 3.15% in heated oil with the 0.378, 0.189, and 0.126 s/v ratios, respectively. The decrease in the amount of polyunsaturated fatty acids was associated with their greater susceptibility to oxidation processes than monounsaturated fatty acids [32].

### 3.2. Imaging Colorimeter

A change in the colour of many food products is often an indicator of their quality [33]. Rapeseed oil is one of the few food materials where changes in the colour may not be significantly correlated with qualitative degradation processes [34]. Hence, the consumer is often unable to assess, even roughly, the actual oil quality, e.g., the degree of rancidity and the negative impact of heat treatment. The criterion of the International Commission on Illumination was used. According to this recommendation, the *ΔE* range of 0–1 means unrecognisable colour changes, the *ΔE* 1–2 range indicates slight changes recognisable only by an expert, and *ΔE* values over 2 denote distinct differences. The calculation procedure are described in the Supplementary Materials A (Section 2. Imaging colorimeter).

**Figure 2:**
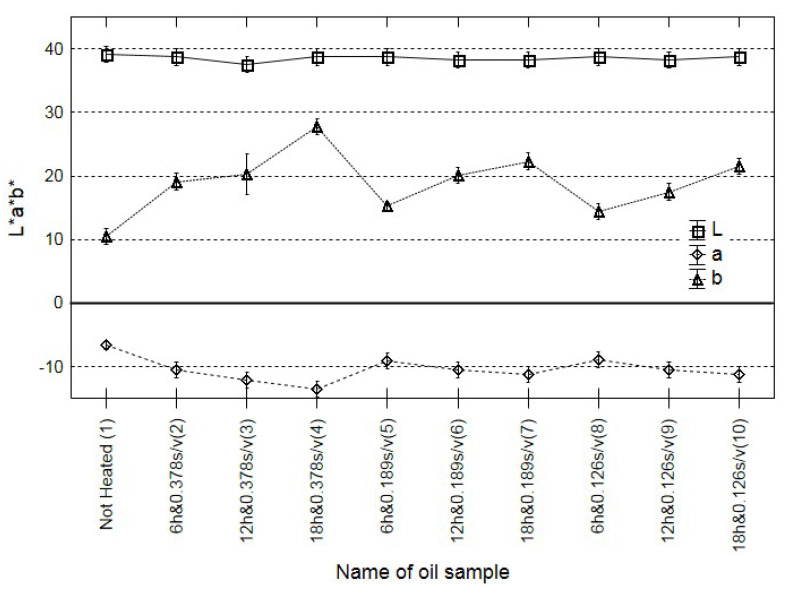
Changes in the colour (L a b) of all oil fried samples and the control.

Similar conclusions can be formulated based on the analysis of changes in the colour. There were no significant differences correlated with the duration of heating, with the ratio of the surface area of oil in contact with oxygen and oil volume, and with the change in the content of polar compounds (Figure 2). Similar results were obtained in previous analyses of cold-pressed rapeseed oil with different quality expressed by the acid value [17]. The present results support the thesis that oil colour may not be an adequate marker of oil quality.

### 3.3. Identification of Major Volatile Compounds—GC-MS Analysis

The oil chromatographic analysis identified a few or several dozen characteristic volatile compounds [35] present in the samples differing in the volume and constant surface of contact with oxygen and subjected to the 6-, 12-, and 18-h frying process determining the oil odour [36]. Table 2 presents the main volatile chemicals in the heat-treated oils and in the control sample as a function of retention time. The greatest levels of volatile compounds were identified in the heat-untreated sample, whereas their decline was determined in the oils subjected to the frying process, as in the case of sunflower oil analysed by Multari [19]. Thermal processing, such as frying, is associated with thermal oxidation and decomposition of oil, resulting in generation of volatile and non-volatile decomposition products [19]. Both types of products are important: volatile compounds influence the flavour of food, whereas non-volatile compounds determine the time of oil frying and the stability of fried food. Non-volatile compounds can also serve as precursors for further deterioration of oil quality. Volatile compounds are primarily responsible for the positive and negative flavours of fried food [37]. In oils heated to frying temperatures, degradation of fatty acids leads to formation of many compounds, e.g., 2,4-nonadienal and 2,4-heptadienal identified in the present study, which are involved in the generation of the odour of deep-fried oil. Another characteristic volatile compound from the alkene group, i.e., *1-decyne*, was identified in all thermally treated samples [38]. These compounds produce the characteristic odours and flavours influencing the taste of frying oil and fried products. A majority of volatile compounds generated in the frying process, i.e., aldehydes, ketones, alkenes, alcohols, acids, esters, hydrocarbons, and furans, are generally undesirable.

### 3.4. Electronic Nose Responses

Table 3 shows two parameters *ΔR/R_max_* and *t_Ratio_* of the response of the electronic nose to the intensity of the odour profile in the analysed oil samples [26]. The *ΔR/R_max_* parameter exhibits a tendency towards intensification of the response with the increasing heating time. Sensor TGS2612 did not follow this trend. The lowest values were recorded for the control sample. The *t_Ratio_* parameter was not correlated with the frying time: it was more or less constant with the highest values in the case of the thermally untreated oil. The ratio of the cleaning time to the reaction time indicates that the former was approx. 4-fold shorter than the latter in the case of the unheated oil. This can be interpreted by the slow adsorption of the molecules on the active surface of the metal oxide semiconductor chemoresistors and their 4-fold faster desorption rate [22,23]. The reaction time in the case of the heated oils was longer than that of the unheated sample.

### 3.5. Principal Component Analysis (PCA)

**Figure 3:**
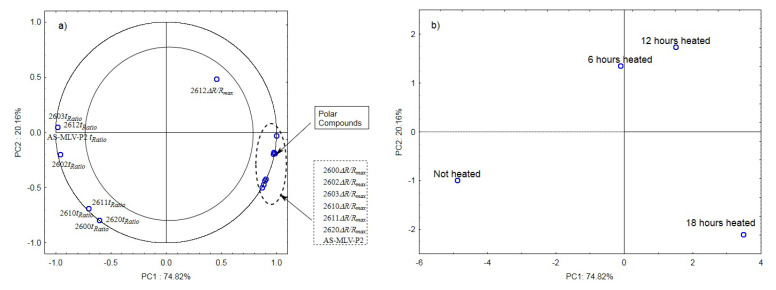
(**a**)—Plot of two principal component loadings (PC1, PC2) from eight sensor readings and polar compounds obtained for heated (0.378 s/v ratio) and control oil samples (*ΔR/R_max_, t_Ratio_*; AS brief names of sensors—AS-MLV-P2; 2600, 2602, 2603, 2610, 2611, 2612, 2620—brief names of sensors Figaro—TGS series); (**b**)—Plot of two principal component scores (PC1, PC2) from eight sensor readings and polar compounds obtained for heated (0.378 s/v ratio) and control oil samples.

The analysis of the principal components for oil samples with the 0.378 s/v ratio of the oxygen contact surface to the volume revealed a positive correlation between the maximum sensor responses ***Δ****R/R_max_* and the level of polar compounds, as shown in Figure 3a. In turn, a negative correlation was found between the *t_Ratio_* times of sensors 2602, 2603, 2612, and AS-MLV-P2 and the level of polar compounds. An exception was the TGS2612 sensor response. Figure 3b shows that the first principal component of PC1 differentiates the heat-treated samples from the control sample.

**Figure 4:**
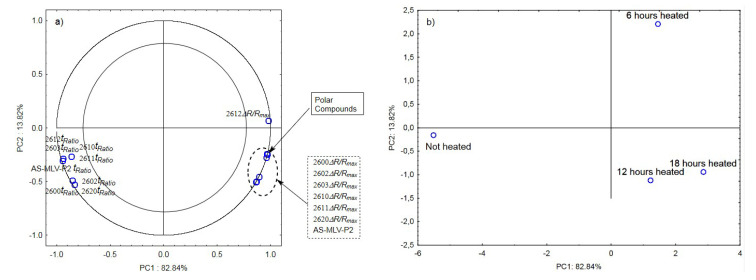
(**a**)—Plot of two principal component loadings (PC1, PC2) from eight sensor readings and polar compounds obtained for heated (0.189 s/v ratio) and control oil samples (*ΔR/R_max_, t_Ratio_*; AS brief names of sensors—AS-MLV-P2; 2600, 2602, 2603, 2610, 2611, 2612, 2620—brief names of sensors Figaro—TGS series); (**b**)—Plot of two principal component scores (PC1, PC2) from eight sensor readings and polar compounds obtained for heated (0.189 s/v) and control oil samples.

The next graphs show the correlation of the maximum sensor responses and time ratio with the content of polar compounds for oil with the 0.189 s/v ratio (Figure 4a) as well as projection of the cases on the PC1 and PC2 planes (Figure 4b). Figure 4a indicates a positive correlation of the maximum responses *ΔR/R_max_* with the changing content of polar compounds and a negative correlation with *t_Ratio_*. As shown in Figure 4b, the first principal component differentiates the heated samples from the control.

**Figure 5:**
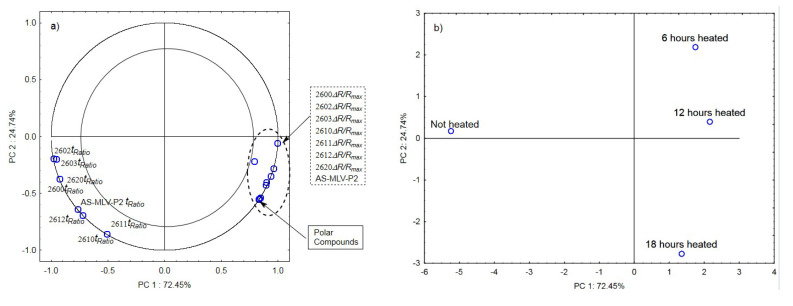
(**a**)—Plot of two principal component loadings (PC1, PC2) from eight sensor readings and polar compounds obtained for heated (0.126 s/v ratio) and control oil samples (*ΔR/R_max_, t_Ratio_*; AS brief names of sensors—AS-MLV-P2; 2600, 2602, 2603, 2610, 2611, 2612, 2620—brief names of sensors Figaro—TGS series); (**b**)—Plot of two principal component scores (PC1, PC2) from eight sensor readings and polar compounds obtained for heated (0.126 s/v) and control oil samples.

Figure 5a,b show the results of the principal component analysis for oils with the 0.126 s/v ratio. Figure 5a is a projection of loadings on the PC1 and PC2 planes, which shows a significant positive correlation between the maximum sensor responses and changes in the content of polar compounds as a function of the frying time and a negative correlation with the time ratio. In this case, the projection of loadings on the PC1 and PC2 planes differentiates the heated samples from the control sample (PC1) and the heating time (PC2) (see Figure 5b).

**Figure 6:**
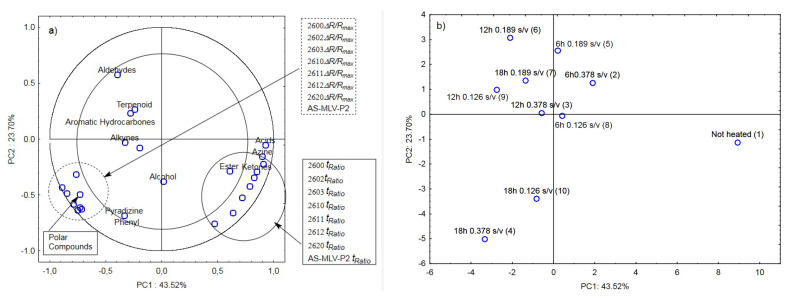
(**a**)—Plot of two principal component loadings (PC1, PC2) from eight sensor readings (*ΔR/R_max_, t_Ratio_*; AS brief names of sensors—AS-MLV-P2; 2600, 2602, 2603, 2610, 2611, 2612, 2620—brief names of sensors Figaro—TGS series), volatile compounds (A-alcohols, AC-acids, E-esters, T-terpenoids, K-ketones, AL-aldehydes, PH-phenyls, ARH-aromatic hydrocarbons, AZ-azines, P-pyradizines, ALK-alkynes) and polar compounds obtained for heated oils: 0.378 s/v ratio; 0.189 s/v ratio; 0.126 s/v ratio and control samples; frying time: 6 h, 12 h, 18 h; (**b**)—Plot of two principal component scores (PC1, PC2) from eight sensor readings, volatile compounds, polar compounds obtained for heated and control samples oil (the same description of names as in Figure 6a).

The last analysis was a collective analysis of nine heated oil samples and the control sample. It was focused on the maximum responses of the electronic nose sensor matrix *ΔR/R_max_* and *t_Ratio_* of the volatile chemical groups [19] (aldehydes, terpenoids, aromatic hydrocarbons, alkynes, alcohols, phenyls, pyradizines, esters, ketones, azines, and acids) and polar compounds. As in the three previous cases, the maximum response *ΔR/R_max_* for the oil samples with the surface-to-volume ratio (s/v ratio) of 0.378 cm^−1^, 0.189 cm^−1^, and 0.126 cm^−1^ was significantly positively correlated with the marker of oil quality change, i.e., the Total Polar Compounds (TPC) level (Figure 6a). The other parameter determined from the e-nose responses, i.e., the ratio of the cleaning time and the reaction time *t_Ratio_*, exhibited a positive correlation with the presence of acids, azines, esters, and ketones (Figure 6a).

Figure 6b shows a projection of the cases (ten oil samples with different surface-to-volume ratios and heating times) on the PC1 and PC2 planes. The first principal component PC1 distinguishes between the unheated and heated oil samples. On the right side of PC1 at the farthest position from the vertical axis is the ‘Not heated’ sample; the samples heated for 6 h are located at a closer position, and the left side of PC1 comprises the other samples heated for 12 and 18 h. This indicates that an electronic nose equipped with eight chemically sensitive MOS sensors is able to not only distinguish oil samples according to their origin [39] and authenticity [40] but also differentiate heated from unheated samples. The device also allows approximate arrangement of oil samples as a function of the heating time. On the other hand, the collective analysis for all ten cases showed that the electronic nose did not differentiate the samples due to the surface-to-volume ratio (s/v ratio) of 0.378 cm^−1^, 0.189 cm^−1^, and 0.126 cm^−1^. This analysis shows that the frying time has a much greater influence on the odor emissions than the surface-to-volume ratio.

## 4. Conclusions

The present investigations demonstrate that oil quality loss as a function of frying time is almost impossible to assess based on changes in the colour of oil. Other chemical methods for determination of total chromanols and total polar compounds efficiently describe changes occurring in oil, but they require higher workload in comparison with the analysis of volatile substances based on the use of the electronic nose. The technique using an e-nose equipped with a matrix of chemically sensitive MOS sensors is a promising tool for rapid non-invasive assessment of changes occurring in oil as a function of frying time. The analysis of the main components showed that, on the one hand, the results of the intensity of odours measured by the electronic nose are correlated with oil quality indicators; on the other hand, the device “is able to distinguish” heat-treated from non-heated oils. The volatile compounds identified in the samples were mainly associated with degradation of fatty acids induced by the heat treatment. Several such substances were detected in this study. The collective analysis for all ten cases showed that the electronic nose did not differentiate the samples due to the surface-to-volume ratio, but this research shows that the frying time has an influence on the on olfactory profile of rapeseed oil.

## Figures and Tables

**Table 1:** Fatty acid composition [%], iodine value (CIV), and total chromanol content [mg/100 g] in refined rapeseed oil heated at 170 °C with different surface-to-volume (s/v) ratios.

	Not Heated	0.378			0.189			0.126		
		6 h	12 h	18 h	6 h	12 h	18 h	6 h	12 h	18 h
Fatty Acid Composition										
C 16:0	4.76 ± 0.00	5.24 ± 0.07	5.34 ± 0.14	5.47 ± 0.02	4.89 ± 0.03	5.04 ± 0.07	5.15 ± 0.10	4.71 ± 0.06	4.77 ± 0.10	4.86 ± 0.17
C 18:0	1.56 ± 0.01	1.53 ± 0.30	1.76 ± 0.00	1.86 ± 0.01	1.57 ± 0.06	1.66 ± 0.02	1.75 ± 0.00	1.61 ± 0.01	1.65 ± 0.00	1.64 ± 0.05
C 18:1	63.83 ± 0.02	67.17 ± 0.30	67.84 ± 0.27	70.13 ± 0.34	65.52 ± 0.28	66.88 ± 0.01	67.82 ± 0.18	65.16 ± 0.08	66.08 ±0.00	66.75 ± 0.08
C 18:2	19.07 ± 0.03	17.22 ± 0.05	16.73 ± 0.58	15.13 ± 0.20	18.21 ± 0.07	17.46 ± 0.04	16.78 ± 0.06	18.42 ± 0.04	17.97 ± 0.07	17.58 ± 0.06
C 18:3	8.29 ± 0.06	6.22 ± 0.15	5.66 ± 0.19	4.65 ± 0.19	7.40 ± 0.21	6.48 ± 0.07	5.92 ± 0.06	7.63 ± 0.03	7.05 ± 0.02	6.63 ± 0.18
C 20:0	0.47 ± 0.01	0.54 ± 0.02	0.56 ± 0.02	0.62 ± 0.01	0.51 ± 0.02	0.53 ± 0.01	0.56 ± 0.02	0.50 ± 0.01	0.51 ± 0.01	0.54 ± 0.01
C 20:1	1.66 ± 0.06	1.69 ± 0.03	1.66 ± 0.05	1.75 ± 0.04	1.53 ± 0.06	1.59 ± 0.06	1.65 ± 0.03	1.56 ± 0.06	1.65 ± 0.03	1.62 ± 0.08
C 22:1	0.26 ± 0.00	0.28 ± 0.02	0.30 ± 0.04	0.29 ± 0.02	0.26 ± 0.02	0.26 ± 0.03	0.29 ± 0.03	0.27 ± 0.03	0.22 ± 0.09	0.29 ± 0.02
C 24:0	0.11 ± 0.02	0.11 ± 0.01	0.15 ± 0.02	0.11 ± 0.05	0.11 ± 0.03	0.09 ± 0.01	0.09 ± 0.04	0.14 ± 0.04	0.09 ± 0.07	0.10 ± 0.01
SFA	6.90± 0.04	7.43 ± 0.26	7.81 ± 0.15	8.05 ± 0.04	7.09 ± 0.04	7.32 ± 0.06	7.54 ± 0.13	6.96 ± 0.06	7.02 ± 0.17	7.13 ± 0.21
MUFA	65.74± 0.04	69.14 ± 0.27	69.80 ± 0.34	72.17 ± 0.35	67.30 ± 0.29	68.74 ± 0.08	69.76 ± 0.20	66.99 ± 0.02	67.96 ± 0.12	68.66 ± 0.01
PUFA	27.36 ± 0.08	23.43 ± 0.15	22.39± 0.48	19.77 ± 0.37	25.61 ± 0.27	23.94 ± 0.07	22.70 ± 0.11	26.04 ± 0.07	25.03 ± 0.05	24.21 ± 0.22
CIV	111.09 ± 0.16	105.37 ± 0.40	103.65 ± 0.50	100.25 ± 0.51	108.63 ± 0.40	106.15 ± 0.14	104.37 ± 0.13	109.31 ± 0.14	107.87 ± 0.15	106.68 ± 0.53
Total chromanols *	82.14 ± 0.56	4.10 ± 0.35	0.33 ± 0.04	0.03 ± 0.03	30.60 ± 1.78	5.53 ± 0.55	1.42 ± 0.26	49.35 ± 1.21	24.36 ± 2.79	2.80 ± 0.47

* Total chromanols is a sum of α-, ß-, γ-, δ-tocopherol and plastochromanol 8.

**Table 2:** Volatile compounds identified by GC-MS analysis of heated oil.

Volatile Compounds									
Not Heated (1)	6 h 0.378 s/v (2)	12 h 0.378 s/v (3)	18 h 0.378 s/v (4)	6 h 0.189 s/v (5)	12 h 0.189 s/v (6)	18 h 0.189 s/v (7)	6 h 0.126 s/v (8)	12 h 0.126 s/v (9)	18 h 0.126 s/v (10)
1.2 * 4(3*H*)-quinazolinone, 2-methyl-3-(2-methylphenyl)	1.2 * acetic acid, trichloro	1.2 *N,N-dimethyl-2*H*-pyran-2-iminium chloride	1.2 *N,N-dimethyl-2*H*-pyran-2-iminium chloride						
1.8 * phenol,3-methyl-4-nitro-, benzenesulfonate (ester)	1.8 * 2-octadec-1-enyloxy-1,1,2,2-tetradeutero ethanol	1.8 * 2-octadec-1-enyloxy-1,1,2,2-tetradeutero ethanol	1.8 * 2-octadec-1-enyloxy-1,1,2,2-tetradeutero ethanol	1.8 * 3,4-dihydrothienyl-(3,4,β)-5-carboxythiol	1.8 * pentasiloxane,1,2,3,3,5,5,7,7,9,9-decamethyl	1.8 * 2-penten-1-ol	1.8 * 2h-pyrrole,2,2-dimethyl-3,5-diphenyl,1-oxide	1.8 * 2-octadec-1-enyloxy-1,1,2,2-tetradeutero ethanol	1.8 * methyl-d3 1-diderterio-2-propenyl ether
3.0 * 4-methoxy-2-(1-phenyletheny)phenol	3.0 * 2-[4-(benzyloxy)phenoxy]-n-(2-nitrophenyl)acetamide	3.0 * 2-[4-(benzyloxy)phenoxy]-n-(2-nitrophenyl)acetamide	3.0 * 2-thiopheneethanol,5-(4,5-dihydro-4,4-dimethyl-2-oxazolyl)-	3.0 * 2-thiopheneethanol,5-(4,5-dihydro-4,4-dimethyl-2-oxazolyl)-					
4.8 * 1h-indole,5-chloro-			4.8 * cyano-[5-(2-hydroxy-acetyl)-pyrrolidin-2-ylidene]-acetic acid tert-butyl ester						
			8.1 * benzenamine, n-(10,11-dihydro-5h-dibenso[a,d]cyclohepten-5-ylidene-,n-oxide		8.1 * 3,4-pyridinedicarboxylic acid,6-(diethylamino)-2-methoxy-,dimethyl ester	8.1 * 3,4-pyridinedicarboxylic acid,6-(diethylamino)-2-methoxy-,dimethyl ester			
	8.4 * 3,4-pyridinedicarboxylic acid,6-(diethylamino)-2-methoxy-,dimethyl ester		8.4 * 3(5)-(4-chlorophenyl)-4-nitroso-5(3)-phenylaminopyrazole	8.4 * (1,3-dimethyl-2-methylene-cyclopentyl)-methanol	8.4 * 1-(2,3-dimethylphenyl)anthracene	8.4 * 1-(2,3-dimethylphenyl)anthracene		8.4 * 1-(2,3-dimethylphenyl)anthracene	
9.0 * 6,9,12-octadecatrienoic acid, methyl ester	9.0 * 2,4-heptadienal,	9.0 * 2,4-heptadienal	9.0 * 2,4-heptadienal,	9.0 * 2,4-heptadienal,	9.0 * 2,4-heptadienal,	9.0 * 2,4-heptadienal,	9.0 * 2,4-heptadienal,	9.0 * 2,4-heptadienal	9.0 * 2,4-heptadienal,
10.8 * cis-9,10-dimethylanthracene-9-10-diol									
12.0 * 4,5-secocholest-8-en-3-one, 5-ethylidene-14-methyl	12.0 * 1-decyne	12.0 * 1-decyne	12.0 * 1-decyne	12.0 * 1-decyne	12.0 * 1-decyne	12.0 * 1-decyne	12.0 * 1-decyne	12.0 * 1-decyne	12.0 * 1-decyne
17.6 * 13-heptadecyn-1-ol									
18.0 * 2-methyl-3-ethoxycarbonyl-4-phenyl-5-oxoindeno [1,2-b]pyridine oxime	18.4 * cycloheptane-pentanoic acid, 1-nitro-β,2-dioxo,phenylmethyl ester	18.4 * endo-dicyclopentadiene dioxide	18.4 * tricyclo[6,3,3,0] tetradec-4-ene-10-dione	18.4 * 2,4-nonadienal,	18.4 * 2,4-nonadienal,	18.4 * 2,4-nonadienal,	18.4 * 2,4-nonadienal,	18.4 * 2,4-nonadienal,	18.4 * 2,4-nonadienal
26.0 * 2,3-diethyl-1,5,7-trimethoxyindenone			26.0 * 1,2-benzenediccarboxylic acid, diethyl ester						
31.5 * stearic acid, 3-(octadecyloxy)propyl ester		31.5 * 1,2-benzenediccarboxylic acid, butyl octyl ester	31.5 * 2,7-diphenyl-1,6-dioxopyridazino[4,5-2,3]pyrrolo[4,5-δ]pyridazine		31.5 * 4h-1-benzopyran-4-one,2-(3,4-dihydroxyphenyl)-3,5-dihydroxy-7-methoxy				

* Rt—retention time.

**Table 3:** Responses of metal oxide semiconductor chemoresistors to the odour profile of heated oil.

	TGS2602	AS-MLV-P2	TGS2603	TGS2612	TGS2610	TGS2611	TGS2620	TGS2600
	*Δ* *R/R_max_*							
Not heated (1)	0.17 ± 0.01	0.025 ± 0.0	0.08 ± 0.00	0.01 ± 0.00	0.03 ± 0.00	0.03 ± 0.00	0.05 ± 0.01	0.05 ± 0.00
6 h 0.378 s/v (2)	0.5 ± 0.01	0.22 ± 0.01	0.3 ± 0.01	0.01 ± 0.00	0.1 ± 0.01	0.1 ± 0.01	0.22 ± 0.01	0.24 ± 0.01
12 h 0.378 s/v (3)	0.8 ± 0.01	0.4 ± 0.01	0.4 ± 0.01	0.1 ± 0.00	0.2 ± 0.01	0.19 ± 0.01	0.32 ± 0.01	0.37 ± 0.01
18 h 0.378 s/v (4)	1.11 ± 0.03	0.57 ± 0.02	0.5 ± 0.02	0.03 ± 0.00	0.4 ± 0.01	0.37 ± 0.02	0.82 ± 0.03	0.87 ± 0.02
6 h 0.189 s/v (5)	0.52 ± 0.02	0.25 ± 0.01	0.24 ± 0.01	0.02 ± 0.00	0.1 ± 0.01	0.1 ± 0.01	0.2 ± 0.01	0.22 ± 0.01
12 h 0.189 s/v (6)	0.65 ± 0.01	0.34 ± 0.01	0.3 ± 0.01	0.02 ± 0.00	0.17 ± 0.01	0.17 ± 0.01	0.39 ± 0.01	0.43 ± 0.01
18 h 0.189 s/v (7)	0.77 ± 0.01	0.36 ± 0.01	0.33 ± 0.01	0.02 ± 0.00	0.19 ± 0.01	0.19 ± 0.01	0.44 ± 0.02	0.5 ± 0.02
6 h 0.126 s/v (8)	0.6 ± 0.01	0.34 ± 0.01	0.25 ± 0.01	0.015 ± 0.00	0.2 ± 0.01	0.2 ± 0.01	0.42 ± 0.02	0.45 ± 0.02
12 h 0.126 s/v (9)	0.92 ± 0.02	0.41 ± 0.01	0.61 ± 0.02	0.015 ± 0.00	0.22 ± 0.01	0.24 ± 0.01	0.55 ± 0.02	0.6 ± 0.02
18 h 0.126 s/v (10)	0.94 ± 0.02	0.45 ± 0.02	0.4 ± 0.02	0.015 ± 0.00	0.3 ± 0.01	0.28 ± 0.01	0.8 ± 0.02	0.86 ± 0.02
	*t_Ratio_*							
Not heated (1)	0.24 ± 0.01	0.24 ± 0.02	0.24 ± 0.01	0.24 ± 0.02	0.18 ± 0.01	0.18 ± 0.01	0.24 ± 0.02	0.24 ± 0.02
6 h 0.378 s/v (2)	0.2 ± 0.01	0.16 ± 0.01	0.16 ± 0.01	0.16 ± 0.01	0.1 ± 0.01	0.1 ± 0.01	0.15 ± 0.01	0.15 ± 0.01
12 h 0.378 s/v (3)	0.2 ± 0.01	0.16 ± 0.01	0.16 ± 0.01	0.16 ± 0.01	0.1 ± 0.01	0.1 ± 0.01	0.12 ± 0.01	0.12 ± 0.01
18 h 0.378 s/v (4)	0.16 ± 0.01	0.11 ± 0.01	0.11 ± 0.00	0.11 ± 0.01	0.11 ± 0.01	0.11 ± 0.01	0.19 ± 0.01	0.19 ± 0.01
6 h 0.189 s/v (5)	0.16 ± 0.01	0.11 ± 0.01	0.11 ± 0.00	0.08 ± 0.00	0.08 ± 0.01	0.08 ± 0.00	0.11 ± 0.01	0.11 ± 0.00
12 h 0.189 s/v (6)	0.2 ± 0.01	0.16 ± 0.01	0.16 ± 0.01	0.12 ± 0.01	0.12 ± 0.01	0.12 ± 0.01	0.16 ± 0.01	0.16 ± 0.01
18 h 0.189 s/v (7)	0.2 ± 0.01	0.12 ± 0.01	0.12 ± 0.00	0.12 ± 0.01	0.08 ± 0.01	0.08 ± 0.00	0.16 ± 0.01	0.16 ± 0.01
6 h 0.126 s/v (8)	0.16 ± 0.01	0.09 ± 0.00	0.09 ± 0.01	0.04 ± 0.00	0.07 ± 0.00	0.07 ± 0.00	0.11 ± 0.01	0.11 ± 0.01
12 h 0.126 s/v (9)	0.16 ± 0.01	0.12 ± 0.01	0.13 ± 0.00	0.09 ± 0.00	0.09 ± 0.00	0.09 ± 0.00	0.13 ± 0.01	0.12 ± 0.01
18 h 0.126s/v (10)	0.18 ± 0.01	0.20 ± 0.01	0.13 ± 0.01	0.12 ± 0.02	0.11 ± 0.01	0.11 ± 0.00	0.17 ± 0.01	0.16 ± 0.01

## Data Availability

Not applicable.

## Supplementary Materials

The following are available online at https://www.mdpi.com/1424-8220/21/1/303/s1, Figure S1: Scheme of a typical sensorgram for MOS sensors with a ratio of reaction times of *T_R_* and *T_CL_* marked on the graph; Figure S2: Plot of two principal component scores (PC1, PC2) from eight sensor readings and polar compounds obtained for heated (0.378 s/v ratio) and control oil samples determined on the basis of three repetitions of measurements; Figure S3: Plot of two principal component scores (PC1, PC2) from eight sensor readings and polar compounds obtained for heated (0.189 s/v) and control oil samples determined on the basis of three repetitions of measurements; Figure S4: Plot of two principal component scores (PC1, PC2) from eight sensor readings and polar compounds obtained for heated (0.126 s/v) and control oil samples determined on the basis of three repetitions of measurements. PCA analysis based on all experimental data.
