# Supplementary material for: Identification of the Olfactory Profile of Rapeseed Oil as a Function of Heating Time and Ratio of Volume and Surface Area of Contact with Oxygen Using an Electronic Nose
Source: Sensors (Basel). 2021 Jan 5;21(1):303. doi: 10.3390/s21010303 (PMC7794714; doi:10.3390/s21010303)
Supplement: Supplementary file 1 [file sensors-21-00303-s001.pdf]

Supplementary materials to

Identification of profile of volatile compounds in rapeseed oil as a function of heating time and ratio of volume and surface area of contact with oxygen using electronic nose

Robert Rusinek*[1], Dominik Kmiecik[2], Marzena Gawrysiak-Witulska[3], Urszula Malaga-Toboła[4], Sylwester Tabor[4], Pavol Findura[5], Aleksander Siger[6], Marek Gancarz[1,4]

[1]Institute of Agrophysics Polish Academy of Sciences, Doświadczalna 4, 20-290 Lublin, [2]Department of Gastronomy Science and Functional Food, Faculty of Food Science and Nutrition, Poznan, University of Life Sciences, Wojska Polskiego 31, 60-634 Poznan, Poland
[3]Institute of Food Technology of Plant Origin, Faculty of Food Science and Nutrition, Poznań University of Life Science, Wojska Polskiego 28, 60-637 Poznan, Poland, [4] Faculty of Production and Power Engineering, University of Agriculture in Krakow, Balicka 116B, 30-149 Kraków Poland,[5]Department of Machines and Production Biosystems, Faculty of Engineering, Slovak University of Agriculture in Nitra, Hlinku 2, Nitra, 949 76, Slovakia,[6]Department of Food Biochemistry and Analysis, Faculty of Food Science and Nutrition, Poznań University of Life Sciences, Wojska Polskiego 31, 60-634 Poznań, Poland

* Corresponding author: r.rusinek@ipan.lublin.pl (R.Rusinek); Tel.: +48 81 744 50 61; fax +48 744 50 67.

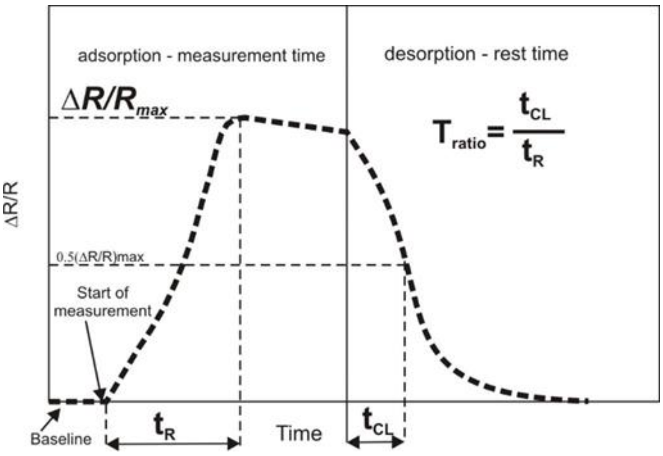

Figure S1. Scheme of a typical sensorgram for MOS sensors with a ratio of reaction times of T_R and T_{CL} marked on the graph

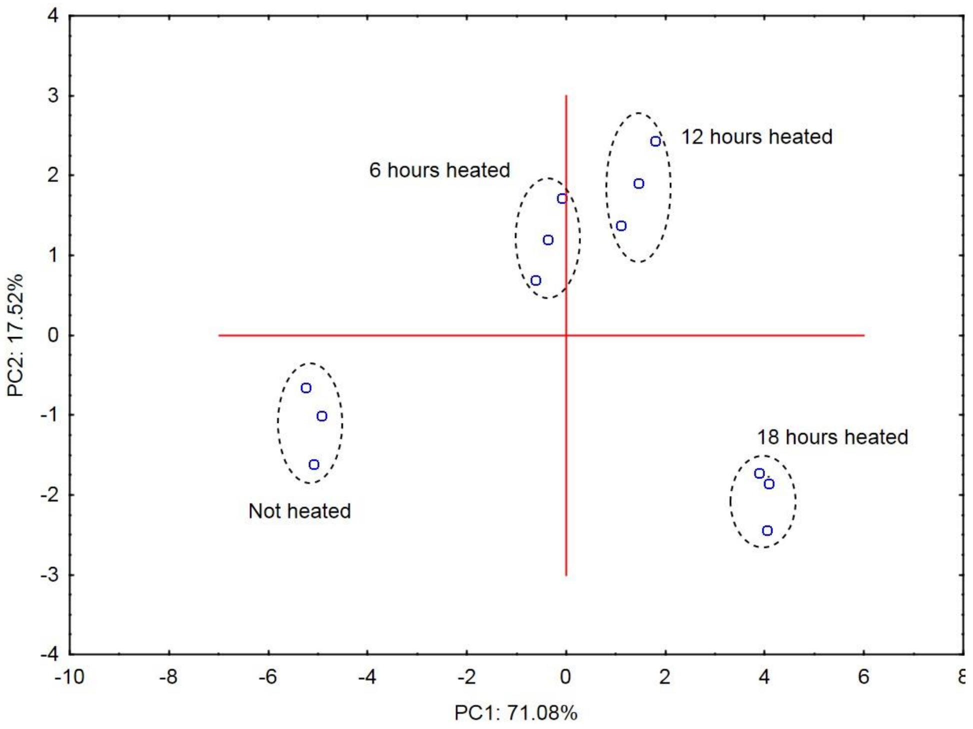

Figure S2. Plot of two principal component scores (PC1, PC2) from eight sensor readings and polar compounds obtained for heated (0.378 s/v ratio) and control oil samples determined on the basis of three repetitions of measurements.

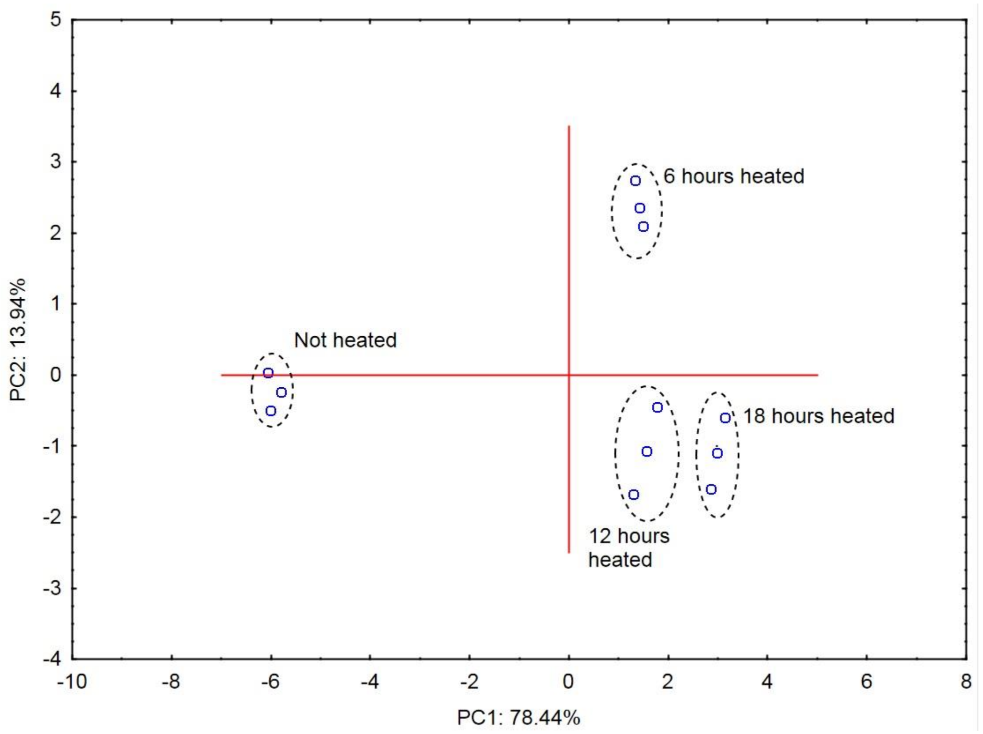

Figure S3. Plot of two principal component scores (PC1, PC2) from eight sensor readings and polar compounds obtained for heated (0.189 s/v) and control oil samples determined on the basis of three repetitions of measurements.

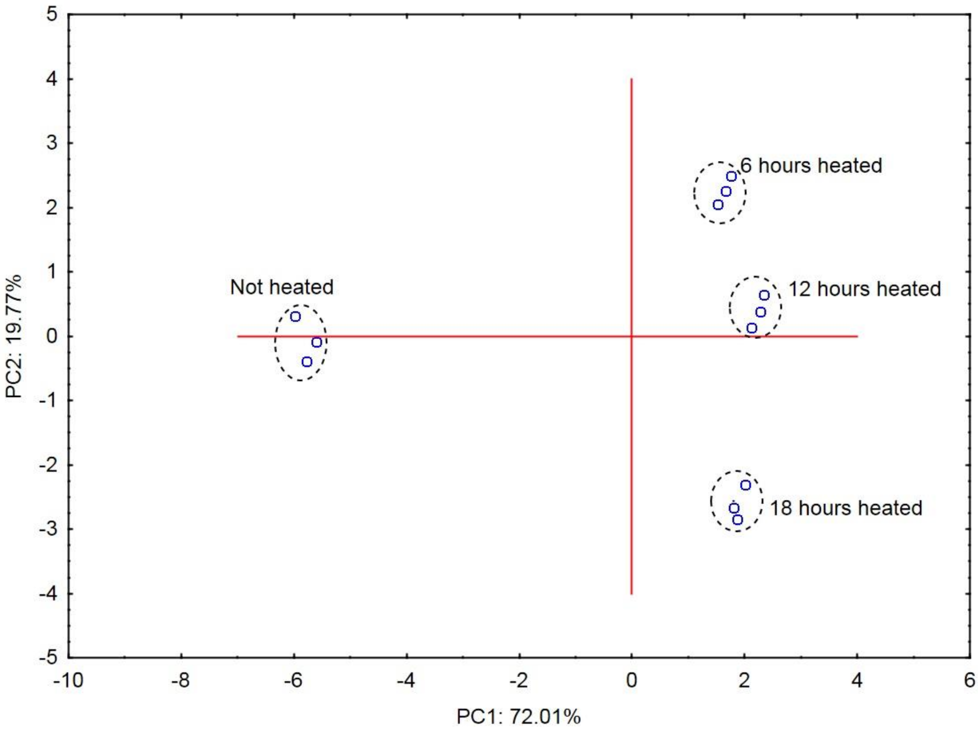

Figure S4. Plot of two principal component scores (PC1, PC2) from eight sensor readings and polar compounds obtained for heated (0.126 s/v) and control oil samples determined on the basis of three repetitions of measurements.

1.Electronic nose procedure

The Agrinose device designed and constructed at the Institute of Agrophysics of the Polish Academy of Sciences in Lublin was used in the study. It has a matrix of eight MOS sensors. Table 1S presents the types and technical data of Agrinose sensors. Seven of them (TGS type) were produced by Figaro Engineering (Japan) and one by Ams (USA). A measurement cycle according to the sample protocol consisted of 10 s baseline purge, 40 s sample draw-in, 5 s laboratory air purge and 140 s sample purge. Analog signals were converted to digital signals by means of software of Agrinose. Obtained sensorgrams were converted to the ∗.xls format and analysed using software Statistica (version 12.0, StatSoft Inc., USA).

Table S1. Technical data of Agrinose sensors

Type	Description	Detecting range (ppm)
TGS2600 - B00	General air contaminants, hydrogen and carbon monoxide	1 - 3 (H_2)
TGS2610 - C00	LP gas, butane	500 - 10 000
TGS2602 - B00	Ammonia, Hydrogen sulfide (high sensitivity to VOC and odorous gases)	1 - 30 (EtOH)
TGS2611 - C00	Natural gas, methane	500 - 10 000
TGS2603 - B01	Odors generated from spoiled foods	1 - 10 (EtOH)
TGS8100 MEMS	Cooking odors, gaseous air contaminants	1 – 30 H_2
TGS2620 - C00	Solvent vapours, volatile vapors, alcohol	50 - 5 000
AS – MLV - P2	CO, butane, methane, ethanol, hydrogen. Specifically designed for volatile organic compounds (VOCs)	10 - 10 000

Agrinose was previously used to evaluate bread spoilage, rancidity of edible oils, and seed spoilage and for control of baking. In comparison with the previous studies, the device was modified by replacing sensor TGS2611 E00 (sensitivity to natural gas) with sensor TGS2603 for detection of food spoilage, which significantly improved the sensitivity of the device in identification of VOCs in biological materials.

In contrast to common descriptions of the odor profile, a new three-parameter method for generation of the smellprint was used in the present study (Fig. 1S). To date, the maximum sensor response $\Delta R/R_{max}$, i.e. the maximum value of the change in e.g. the resistance of MOS or CP (conducting polymer) sensors, has been most commonly used for generation of smellprints. The authors developed a new method for generation of smellprints based on two additional parameters: the time until achievement of the maximum response - the response time T_R and the cleaning time T_{CL}, which is the time of removal of molecules from the sensor's active surface, i.e. the

time from achievement of the maximum response $\Delta R/R_{max}$ to half of its value. The parameters established in the study were dependent on the type of volatile substances contained in the odor profile and the intensity of emissions of compounds. A new odor intensity factor was used, i.e. the ratio of the reaction times T_{ratio} (Fig. 1). Its physical interpretation is based on the assumption that the greater the value is than zero, the more intense the interaction of volatile molecules with the active surface is.

2. Imaging colorimeter

The difference in the colours in the CIE space was determined with the use of the following formula:

$$\Delta E=[(\Delta L^*)2 +(\Delta a^*)2 +(\Delta b^*)2]1/2$$

where:

*L** – brightness (L values ranging from 0 (black) to 100 (white),

*a**– value on the red and green axis in the range of *a* ± 120,

*b** – value on the yellow and blue axis the range of *b* ± 120

3. Gas chromatography-mass spectrometry (GC-MS) analysis

The GC-MS analyses were carried out using a Trace GC Ultra gas chromatograph (ThermoFisher Scientific, USA) coupled with an ITQ 1100 mass spectrometer (ThermoFisher Scientific, USA) following the procedure described by Gancarz et al., 2020. Volatile compounds were collected from the headspace by solid phase micro-extraction (SPME). SPME fibre 50/30 µm Divinylbenzene/Carboxen/Polydimethylsiloxane (DVB/CAR/PDMS), Stableflex (2cm) 24Ga (Sigma Aldrich, Poland), was used for the chromatographic analyses. The fibre with the adsorbent was placed for 30 minutes in a measurement chamber with a mixture of volatile organic compounds emitted by the oil through a special valve. Then, it was transferred into a GC injector for 5 minutes for desorption of volatile organic compounds. The injection port equipped with a 0.75-mm i.d. liner was maintained at 250°C in the splitless mode. A Zebron ZB-5Msplus Capillary GC 30m x 0.25mm x 0.25µm capillary column was used in the analysis. The following temperature program was established: initial temperature of 60°C for 5 min increasing from 60 to 250°C at 5°C/min and from 250 to 270°C at 10°C/min, with the final temperature maintained for

5 min. A constant helium flow rate was kept at 2.2 ml/min. The temperature of the transfer line and the ion source was 280°C. The electron ionisation (EI +) mode with an electron energy value of 70 eV was applied. The mass spectrometer collected data in the full scan mode (scan ranges: 35–390). The procedure was also described in other publications by the authors.
